# A Molecular–Structure Hypothesis

**DOI:** 10.3390/ijms11114267

**Published:** 2010-11-01

**Authors:** Jan C. A. Boeyens

**Affiliations:** Unit for Advanced Study, University of Pretoria, Lynnwood Road, Pretoria, South Africa; E-Mail: jan.boeyens@up.ac.za; Tel.: +27-12-420-4528; Fax: +27-12-362-5288

**Keywords:** molecular shape, self-similarity, golden mean, logarithmic spiral, hypercomplex number, quaternion, general covalence, fibonacci phyllotaxis, solar system, atomic structure, quantum potential, space-time topology

## Abstract

The self-similar symmetry that occurs between atomic nuclei, biological growth structures, the solar system, globular clusters and spiral galaxies suggests that a similar pattern should characterize atomic and molecular structures. This possibility is explored in terms of the current molecular structure-hypothesis and its extension into four-dimensional space-time. It is concluded that a quantum molecule only has structure in four dimensions and that classical (Newtonian) structure, which occurs in three dimensions, cannot be simulated by quantum-chemical computation.

## 1. Introduction

Early suggestions that molecules have three-dimensional structures were greeted with derision, and recent efforts to simulate such structures quantum-mechanically have all failed. Apart from experimental success to map atomic networks in the solid state, the definition of invariant shapes, which are intrinsic to molecules, remains shaky. The inherent symmetry of molecular spectra has never been linked with theoretical certainty to rigid structure and the molecular-structure hypothesis remains essentially unsubstantiated, although millions of undisputed molecular connectivities are based directly on these unproved notions of molecular structure and symmetry.

The contentious issue is whether any form of molecular structure can be defined quantum-mechanically at all. The essential problem was put into eloquent perspective many years ago by Claverie [1], stating that:

“[a]lthough a molecule, considered as a set of [...] nuclei and electrons, [...] belongs to the domain of microphysics, it is well-known that [...] the behaviour of its nuclei is treated either through quantum or through classical methods according to the subject of investigation: [...] quantum treatment is used for dealing with vibrational and rotational spectra, while classical reasoning is most frequently used for dealing with conformational analysis [and] reaction mechanisms [...]. [I]t will not be very easy to rebuild some connection [...] from quantum theory toward classical theory [...] and this is a somewhat ridiculous situation since [...] classical models work perfectly for most macroscopic phenomena [...]. [S]ome kind of quantum–classical connection is strongly needed”.

The author reaches the conclusion that

“. . . *quantal* structure manifests its[e]lf only when some measurement takes place, while, according to the notion of *classical* structure, the molecule could be considered a set of point-like nuclei endowed with a well-defined position at any point, independently of any measurement”.

To my mind this is a weak, and even self-contradictory, distinction between a molecule and itself. The author concludes that

“. . . accounting for the classical behaviour [...] is a genuinely non-trivial and essentially unsolved problem which deserves specific and adequate investigation”.

As recommended by a referee I insert the following brief historical account of how the debate on molecular structure has developed [2]:

“Since the late 1970s theoretical chemists, who worked hard on the development of quantum chemical models for chemical purposes, also began to question the naive reductionist, albeit common, view among western philosophers of science, according to which chemical concepts and laws could simply be derived from quantum mechanical principles. Guy Woolley, in a seminal paper [3], argued that the concept of chemical structure cannot be deduced from quantum mechanics. Hans Primas [4] devoted a whole book to the issue of reductionism, arguing that quantum mechanical holism does not allow the derivation of statements about chemical objects without further assumptions. Giuseppe Del Re and Christoph Liegener considered chemical phenomena to lie on a higher level of complexity that emerges from but does not reduce to the quantum mechanical level [5,6].”

The questionable physical grounds for the structure hypothesis are now re-examined and the conjecture that, like other objects in the universe, molecules acquire their shape in harmony with four-dimensional space-time curvature, is also explored.

## 2. Results and Discussion

### 2.1. Cosmic Self-Similarity

The widespread perception that all structures in the cosmos share a common design principle was given some theoretical content by Oldershaw’s statement of cosmic self-similarity [7]. The idea of self-similarity is closely related to fractal geometry and the golden ratio as expressed in the properties of the golden logarithmic spiral. Oldershaw distinguishes between three hierarchical structures at the atomic or subatomic, stellar or substellar and galactic scales. The basic premise is that the fractal nature of the universe leads to self-similarity, or invariance with respect to scale transformation, in which small parts of a structure have geometrical properties that resemble the whole structure or large parts thereof. The most familiar example of self-similarity is the Mandelbrot set and the logarithmic spiral is the only smooth curve that is self-similar at all scales.

Self-similarity between objects on different scales implies the existence of a mathematical operation which is equally effective on different cosmic scales and which operates on symmetry elements in any number of dimensions from one to four. A *quaternion* is such an object, whose operation is best understood in terms of rotation in the complex plane, described by the complex number

$$(a,b)=a+ib\equiv r(\text{cos}\theta +i\text{sin}\theta )=re^{i\theta }$$

which is isomorphic with the matrix $\left(\begin{array}{cc} a & -b\\ b & a \end{array}\right)$, e.g., $\left(\begin{array}{cc} \text{cos}\theta & -\text{sin}\theta \\ \text{sin}\theta & \text{cos}\theta \end{array}\right)$, under addition and multiplication. For example, the complex number *i* is represented by the matrix $\left(\begin{array}{cc} 0 & -1\\ 1 & 0 \end{array}\right)$ that corresponds to a counter-clockwise rotation of π/2 about the origin.

**Figure 1.**
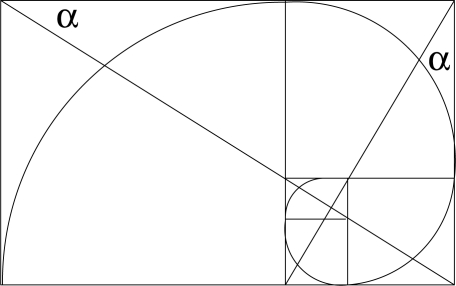
The golden logarithmic spiral. tan α = τ.

The locus of a continuously rotating point is a circle. By also allowing continuous dilatation of the radius, the locus becomes an equiangular, or logarithmic spiral, best known in the classical form

(1) $$r=Ae^{\theta \text{cot}\varphi }$$

where *A* and *φ* are constants. It is common practice to set *A* = 1 to obtain a unit spiral. For *φ* = 72.83° the spiral *r* = *Ae*^*θτ*/2^ is virtually identical to a golden spiral, which develops on removing a square (gnomon) from a golden rectangle and continuing the process indefinitely on the newly created golden rectangles of diminishing size, as shown in Figure 1. The inscribed golden spiral converges to the intersection of diagonals.

More generally, in terms of the complex number *a* + *ib*:

$$r=Ae^{(a/b)\theta },\;\;\;\;\;b\neq 0$$

It is instructive to note that rotation in the complex plane has no counterpart in three dimensions, as first discovered by William Hamilton. The direction of a rotation axis in three dimensions is perpendicular to the complex plane and provides no extra information about the rotation. An equivalent conclusion is that three-dimensional complex numbers are undefined. The simplest hypercomplex number, defined in four dimensions, is known as a quaternion:

q=a+ib+jc+kd

where *i*, *j*, *k* are generalizations of $\sqrt{-1}$, such that

$$\begin{array}{l} i^{2}=j^{2}=k^{2}\\ ij=k,\;\;jk=1,\;\;ki=j;\;\;ji=-k,\;\;kj=-1\;\;ik=-j \end{array}$$

and *q*^2^ = *a*^2^ + *b*^2^ + *c*^2^ + *d*^2^. A four-dimensional rotation is now defined by the quaternion

$$Qe^{\theta (i\alpha +j\beta +k\gamma )}=Q\{\text{cos}\theta +\text{sin}\theta (i\alpha +j\beta +k\gamma )\}$$

Whereas a complex number is defined by a vector and a phase, a quaternion is specified by a *tensor* and a *versor*.

The demonstration that both Lorentz transformation and quantum spin are the direct result of quaternion rotation implies that all relativistic and quantum structures must have the same symmetry. This is the basis of cosmic self-similarity. The observation that the golden mean features in many self-similarities is interpreted to show that τ represents a fundamental characteristic of space-time curvature. The existence of antimatter and the implied CPT symmetry of space-time favours closed metric-free projective geometry with involution; the only topology that automatically generates the gauge invariance that links quantum mechanics to the electromagnetic field [8]. This topology is consistent with constant space-time curvature, locally distorted by large gravitating masses. It seems reasonable to assume that the logarithmic spiral (1) follows the general curvature in two-dimensional projection, characteristic of stable structures and growth patterns in tangent Euclidean space. In four-dimensional space-time the curvature is more appropriately described by a formula such as

$$\rho (x^{\mu })=Ae^{(a/\sqrt{b^{2}+c^{2}+d^{2}})\theta }$$

which describes spherical rotation in quaternion notation.

For our purpose all structures which can be related unequivocally in terms of the golden ratio and/or a golden spiral are assumed universally self-similar. This includes the packing of nucleons, nuclide periodicity, general covalency, nanostructures, botanical phyllotaxis, biological growth, tropical hurricanes, gaps in the asteroid belt, the distribution of moons, rings and planets in the solar system, eddies in globular clusters and the structure of spiral galaxies. An obvious gap in this hierarchy occurs at the level of chemical molecules, although scale symmetries [9] and code regularities [10] have been reported for DNA structures. Self-similarity within series of dendrimers has been observed. However, no mechanism to explain these phenomena at the molecular level has been proposed.

### 2.2. Documented Examples

#### Nucleon Packing

The lowest level at which golden-ratio symmetry has been mooted is in the packing of neutrons and protons in atomic nuclei. The direct evidence is that the fractional ratio *Z*/(*A* − *Z*) of protons to neutrons, for stable (non-radioactive) nuclides, which decreases with mass number, converges to $\tau =\frac{1}{2}(\sqrt{5}-1)=0.61803\ldots$ . . . from an initial value of unity for low mass number. This ratio occurs naturally as the limiting quotient, *n*/(*n* + 1), of successive Fibonacci numbers in the series:

$${\text{F}}_{n}=1\;\;\;\;\;1\;\;\;\;\;2\;\;\;\;\;3\;\;\;\;\;5\;\;\;\;\;8\;\;\;\;\;13\ldots n\;\;\;\;n+1\ldots$$

The space-filling two-dimensional spiral arrangement of growing seedbuds can always be traced along secondary spiral arms with *n* and *n* + 1 units in opposite directions. A similar three-dimensional distribution of protons and neutrons would explain [11] the convergence from 1 to τ. It leaves a surface excess of protons, *x_e_* = *Z* − *N*τ, which converges to zero at *Z* = 102, *N* = 165, *A* = 267. These numbers represent limiting values for 100 natural elements and 300 nuclides.

Scatter plots of *x_e_* *vs A*, *N* or *Z* define a periodic function that arranges the 264 stable nuclides in an 11 × 24 matrix which contains the magic numbers of nuclear physics and the periodic table of the elements as subsets. The corollary is that the periodic table appears as a nuclear property, independent of the extranuclear distribution of electrons as traditionally assumed. All of chemistry is then seen to emerge as a topological property of space-time or the vacuum.

#### General Covalence in Molecules

Molecules are the product of atomic associations. Their formation is best understood for the simplest type of molecule, consisting of two identical atoms. The obvious interpretation is to ascribe the association to a redistribution of extranuclear electrons in the field of two nuclei and stabilization of the diatomic unit by electromagnetic interaction. In order to interact, two atoms need to be activated. If, in the process, an electron reaches the ionization energy level of the atom it is decoupled from its nucleus and free to associate with another, similarly activated, atom. Such an activated atom is said to be in the valence state.

**Figure 2.**
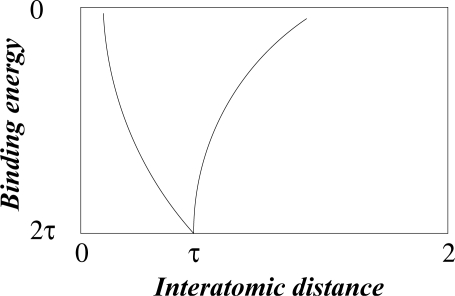
Diatomic covalence curve in dimensionless units.

The redistribution of charge that stabilizes the diatomic molecule is readily simulated by the quantum-mechanical method, first developed by Heitler and London, to model the formation of a hydrogen (H_2_) molecule, or alternatively by classical electrostatics, using point charges to model electron density and monopositive atomic cores in the calculation of dissociation energy as a function of interatomic distance. This relationship, in dimensionless units, valid for all homonuclear diatomic molecules, has the simple form shown in Figure 2 [12].

It fits precisely into a golden rectangle and reaches maximum binding energy of 2τ at an interatomic distance of τ. This result is interpreted to mean that the minimum space needed for a pair of electrons to co-exist is limited by the golden ratio as a universal property of space-time. This *exclusion principle* was first postulated by Pauli on empirical grounds to account for the observed fine structure of atomic spectra.

In chemical terminology the interaction is known as a covalent bond, with maximum strength, like the maximum charge density, conditioned by the golden ratio. If this means that covalent interaction depends on the golden ratio and on the topology of space-time, it is more than likely that the shape of covalent molecules should be self-similar with other structures based on the golden ratio. We propose to investigate this likelihood.

#### Fibonacci Phyllotaxis

The best known, but by no means unique, example of Fibonacci phyllotaxis is the distribution of growing seedbuds in a sunflower head. The seedbuds that increase in size from the central growth point are self-similar in shape and arranged along a logarithmic spiral. Although the primary spiral is not all that obvious, the secondary spiral arms invariably extend over *n* and *n* + 1 seeds and count in opposite sense, where *n* and *n* + 1 are successive Fibonacci numbers. Exactly the same symmetry is observed on a nautilus shell where the separate chambers of increasing size are self-similar, with the logarithmic spiral now the most conspicuous feature.

Variation on this central theme is repeated endlessly in botanical and primitive zoological structures. On closer inspection the golden-ratio curvature that underlies this symmetry is also evident in the bone structures, such as horns and tusks of higher zoological species. Interspecies variation as observed in the fossil record can also be generalized in terms of this same symmetry [13] and we now postulate that biological growth develops on a template that reflects both space-time topology and golden-ratio symmetry. Again, we cannot ignore the likelihood that self-similar features of chemical molecules underpin this biological regularity.

#### The Solar System

All numerical regularities that had been observed in the arrangement of heavenly bodies in the solar system were recently shown [14] to arise from the same symmetries that occur in biological structures.

In botany the arrangement of leaves on a stalk is optimized by the requirement of equal exposure to sunlight, which is achieved by avoiding direct overlap of leaves at different levels on the stalk. The theoretically most efficient and botanically preferred arrangement corresponds to the emergence of a new leaf at an angle of 2πτ^2^ ≡ 137.5° with respect to the previous one. This is the angle that divides a circle in golden ratio: 2πτ^2^/2πτ = 2πτ/2π = τ; τ^2^ + τ = 1.

**Figure 3.**
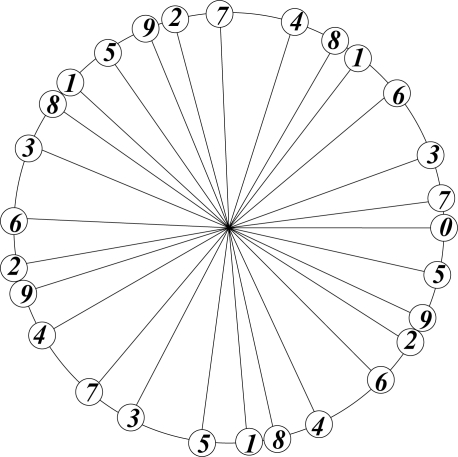
Optimal arrangement of leaves on a stalk.

The arrangement of successive points, generated with this *divergence* angle, starting counterclockwise from 0, and labelling three points per cycle, is shown in Figure 3.

This procedure may be continued indefinitely without causing exact overlap, irrespective of the number of cycles. This is a feature of the irrational nature of τ.

In the case of satellite accretion along a spiral, a different criterion determines the optimal divergence angle. Compared to the botanical divergence angle of 2πτ^2^, which is an area effect, competition for intermediate material along a spiral arm is linear. The preferred divergence angles for planets and moons are multiples of π/5 ≃ πτ/3.

#### Atomic Structure

We now show that the distribution of extranuclear electrons that surround an atomic nucleus, like leaves on a stalk, moons around a planet, and planets around the sun, is subject to optimization governed by a logarithmic spiral.

The most likely structure of an atom is that of a positively charged massive nucleus surrounded by discreet spherical shells of electrons, in sufficient number to balance the nuclear charge, at characteristic distances from the nucleus. Whereas atomic size is wave-mechanically poorly defined, self-similarity suggests that the spacing between electron shells could relate to some divergence angle measured along a golden spiral. What wave mechanics does show, is that a degeneracy of 2*n* − 1 regulates the number of electrons with the highest angular momentum in a shell of principal quantum number *n* [15]. On this basis a divergence angle for optimal spacing of electron shells may be assumed to vary as 4π/(2*n* − 1).

**Figure 4.**
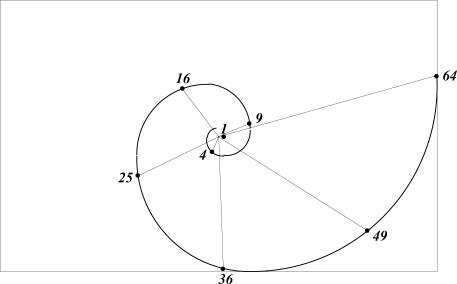
Predicted radii of electron shells as a function of *n*.

Orbital radii, derived graphically, using this prescription, are shown in Figure 4, predicting *r*/*a*_0_ = 1, 4, 9, 16, *etc*., in agreement with the Bohr radii of *r_n_* = *n*^2^*a*_0_. Spectroscopic measurement [16] shows the radii of Rydberg atoms to obey the same formula.

It is of interest to note how the botanical divergence angle solves leaf placement directly; in astronomy it identifies a planar orbit and in the atom, a spherical shell – one, two and three-dimensional problems respectively. Molecular shape will be shown to be a four-dimensional problem. There is no anomaly here: quaternions describe rotations in all sub-spaces of four-dimensional space-time.

### 2.3. Molecular Structure

The periodic table of the elements is correctly predicted in detail as a function of the ratio *Z*/(*A* − *Z*) as it converges from unity to the golden mean. Wave mechanics cannot account for more than a few general features of the observed periodicity and fails to explain the appearance of eight, rather than ten, elements in each transition series.

Electron-pair covalency as a function of the ratio *d*/*r*_0_ of interatomic distance over ionization radius accounts for the observed dissociation energies, *D*, and identifies the exclusion principle at the convergence of *d*/*r*_0_ → τ and *D* → 2τ, in dimensionless units. Wave-mechanical treatment of the problem is limited to a few simple molecules, such as H_2_^+^ and H_2_.

As demonstrated in the previous paragraph, even atomic size, only obliquely related to quantum principles, is directly predicted by a simple model based on self-similarity. We infer with confidence that the shape of isolated molecules is more likely to be revealed by reference to other structures deemed to be a function of space-time curvature.

#### The Structure Hypothesis

The molecular-structure hypothesis is arguably the most controversial issue in theoretical chemistry, but perhaps, the most readily accepted by practising chemists. Not only are three-dimensional structures routinely observed experimentally, by techniques such as X-ray crystallography, but also rationalized, even at junior high-school level, in terms of elementary orbital-hybridization models. Both of these norms are seriously flawed.

In crystallographic analysis an observed electronic charge distribution is assumed to map the inherent geometrical structure of individual chemical species. However, in reality it maps an array of molecules, condensed into periodic alignment and suitably distorted into a space-filling assembly by thermodynamic environment. Occurrence of the exact same conformation, inferred from crystallographic analysis, is hard to substantiate in the liquid state, or in solution. Molecular structure in the gas phase is equally elusive.

Theoretical study of molecular shape is even more uncertain. Quantum observables, as traditionally defined, are associated with suitable operators which generate the eigenvalues that satisfy the wave equation. Such an operator has never been defined for molecular shape, which remains quantum-mechanically undefined. An obvious fall-back position is to use an observed molecular structure as boundary condition in solving for a molecular electronic wave function in the study of a whole range of molecular properties. Except for some success with the smallest of molecules, no results of practical importance have ever been obtained by this method. At a further level of approximation, molecular wave functions, constructed from modified hydrogen-like atomic functions, are used as trial functions to minimize molecular energies within the assumed molecular framework.

With computerized procedures this technique has now been extended into the so-called *ab-initio* LCAO-MO (Linear Combination of Atomic Orbitals – Molecular-Orbital) method, in the belief that the results, including an *optimized* molecular structure, are equivalent to solution of the many-body molecular wave equation. The reason for this strange conviction is that the overwhelming majority of users have no understanding of the commercialized software that they use and trust. The argument is confounded by a number of suspect assumptions:

- The LCAO is based on real functions, whereas quantum-mechanical wave functions are complex;
- Real hydrogenic functions are disallowed by the exclusion principle;
- Minimization of electronic energy, which is a scalar can never produce a three-dimensional shape, which is a vectorial construct;
- The many-body problem cannot be solved, neither classically nor non-classically;
- A rigid molecular structure is at variance with the uncertainty principle; is a classical concept and cannot be optimized quantum-mechanically.

LCAO-MO computations, widely referred to as *Quantum Chemistry*, has a qualitative counterpart, known as orbital-hybridization theory, which is the *de facto* working model of modern chemists and the form in which wave mechanics is handled pedagogically. The sad fact is that this model, which pretends to use rigorous wave-mechanical concepts, vulgarizes quantum theory. It claims Schrödinger’s eigenfunction solution for the hydrogen electron as basis. In order to avoid the use of complex functions, linear combinations, claimed to eliminate imaginary parts, without loss of information, are used instead. These constructs are known as atomic orbitals. An immediate problem is that a degenerate set of such orbitals, considered as eigenfunctions, would have the same quantum numbers *n, l, m_l_*, in violation of the exclusion principle. To avoid this embarrassment quantum numbers are said (see [12], page 64) not to be required any more. This is tantamount to admitting that orbitals have no quantum-mechanical meaning.

The simple explanation of this dilemma is that a linear combination of eigenfunctions does not produce new eigenfunctions, but only rotates the polar axis and hence, also the complex plane. As an example, consider the three-fold degenerate set of, so-called, *p*-functions:

$$p_{l}^{m_{l}}:p^{0}=\frac{z}{r},\;\;p^{1}=\frac{x+iy}{r},\;\;p^{-1}=\frac{x-iy}{r}$$

By linear combination

$$\frac{1}{2}(p^{1}+p^{-1})=\frac{x}{r}\;\;\;\;;\;\;\;\;\frac{1}{2}(p^{1}-p^{-1})=\frac{iy}{r}$$

By choosing a new polar direction along *x* rather than *z*, the set becomes

$$p^{0}=\frac{x}{r},\;p^{1}=\frac{z+iy}{r},\;p^{-1}=\frac{z-iy}{r}$$

a simple rotation of the coordinate axes. Note that *x*/*r* and *z*/*r* cannot co-exist.

Like MO quantum chemistry, the theory of orbital hybridization is a classical model, dressed up in wave-mechanical garb and does not offer a non-classical description of molecular shape.

#### Molecular Shape

The disadvantage of a LCAO is that it eliminates orbital angular momentum as a wave-mechanical variable with eigenvalues proportional to *m_l_*. In so far as *m_l_* = ±1 describes orbital angular-momentum vectors it has the latent ability to decide the relative orientation of interacting chemical fragments. By invoking the principle of optimal quenching of angular momentum during molecule formation, it has been used [12] to predict the structure of small molecules with a single central atom and of torsionally rigid molecules, such as ethylene. The same principle provides a successful physical interpretation of optical activity and the Faraday effect.

This is as far as wave mechanics can account for molecular shape. The question of torsional freedom remains unresolved. Treated classically, by the methods of molecular mechanics, torsional parameters, based on empirical models of non-bonded interaction, are used routinely to reproduce experimentally observed structures. The shape and conformation of an isolated molecule in the vacuum cannot be modelled.

We now make the conjecture that first-neighbour interaction is a function of valence forces and orbital angular momentum, whereas torsional interaction, in the absence of environmental factors, depends on long-range intramolecular effects and the curvature of space-time. We consider a number of relevant examples.

#### Alkanes

An alkane molecule with regular torsion angles of π about all C–C bonds may be assumed to have cylindrical symmetry in the sense that each chain of symmetry-related atoms describes a helix around the central (cylinder) axis. This symmetry is more readily visualized for a molecule such as C*_n_*H*_n_*_+2_Br*_n_*, as shown in the Newman projection down the C*^n^*^+1^– C*^n^* link.

**Figure:**
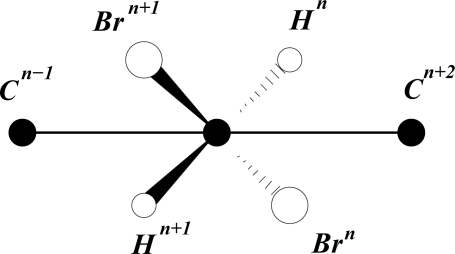


Such structures are known for long-chain hydrocarbons in the crystalline state. However, because of the low level of torsional rigidity, the free molecules are unlikely to assume the same stretched configuration in the gas phase. Because of chaotic entanglement the polymeric structure of polyethylene cannot be used as a guideline. However, to make sense of biological growth structures it seems reasonable to assume a uniform increase of C–C torsion angle, in the absence of distorting influences, such as crystal packing forces. Should the C–C torsion angle deviate slightly from π, *e.g.* as 6/(5τ^2^) or $4\sqrt{\tau }$, the cylindrical axis, at the molecular scale, will curve away, almost imperceptibly from linearity, becoming noticeable only on the macro scale.

#### Polypeptides

The dependence of molecular shape on torsion angle within a chain is a guiding principle in the characterization of structure type in polypeptides and proteins.

**Figure:**
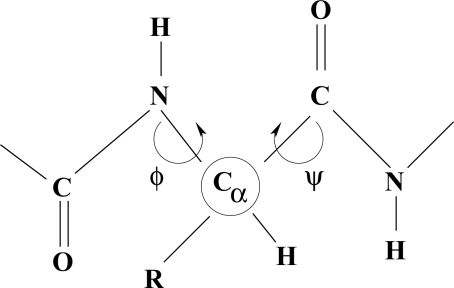


Different conformations, such as an α-helix, depend on characteristic values of the torsion angles φ and ψ. Hydrogen bonds and the nature of R-groups have a decisive influence on the allowed values of these angles. The flexibility of long fibrous structures in collagen is probably responsible for the characteristic curvature of freely-growing horns and tusks of higher vertebrates.

#### Silica Nanostructures

An interesting example of a curved molecular surface that spontaneously adapts to a geometrical arrangement related to the golden ratio has been observed [17]. Patterns, self-similar to botanical phyllotaxis were observed in nanometre-thick silica shells grown on spherical silver cores of 10 micrometre diameter. As the silver core shrinks on cooling, the stress pattern that develops in the silica surface consists of spherules that line up along 5,8 and 13,21 Fibonacci spirals, exactly as in botanical structures.

### 2.4. Discussion

There is a good reason why wave mechanics fails to model molecular systems. By the theory of special relativity we live in a four-dimensional space-time continuum with an interval between events given by

$$s=\sqrt{x_{0}^{2}+x_{1}^{2}+x_{2}^{2}+x_{3}^{3}}$$

in which *x*_0_ = *ict* represents the time coordinate, together with the three usual cartesian coordinates. A transformation between point events is defined by a Lorentz rotation through a complex angle. Such a rotation relates the four coordinates on an equivalent basis, which means that space and time coordinates are not separable in the usual way.

The four-dimensional Laplacian equation

(2) $${□}^{2}\Psi =0$$

which describes potential balance in four-space is also written in the form of a wave equation

$$\nabla^{2}\Psi =\frac{1}{c^{2}}\frac{\partial^{2}\Psi }{\partial t^{2}}$$

which is the basis of Schrödinger’s equation, interpreted to describe matter waves in three-dimensional space. This equation is routinely solved by separating space and time variables. The eigenfunctions, obtained in this way, are formulated as complex functions, whereas solutions to equation (2) are strictly defined in hypercomplex four-dimensional space. A vital aspect of physical reality is lost in the approximation.

As a consequence we find that the nexus between molecular structure and space-time curvature has disappeared. The classical three-dimensional structure, commonly assigned to a molecule, is no more than a crude caricature of the actual quantum structure. That is why so many properties of molecules, such as intramolecular rearrangement, are impossible to reconcile with an assumed classical structure. Even phase transitions, observed to occur in single crystals, often appear to be sterically impossible. That is why the structure of ammonia cannot be correctly formulated in three dimensions [18] and many other molecules cannot be described in terms of Lewis structures.

An extreme case of how molecular structure adapts to environmental factors is observed in the way that the perchlorate ion facilitates crystallization by occupying voids in an otherwise close-packed arrangement [20]. When fitting tightly into a void the ion adopts its familiar tetrahedral structure. In larger cavities it shows up crystallographically as electron density, uniformly spread throughout the entire cavity around the central atom. The effect of temperature [21] confirms that this is not due to static disorder. This result is interpreted to show that the free perchlorate ion is essentially formless and only develops structure under environmental pressure. This phenomenon cannot be addressed classically.

It is beginning to make sense why molecular phenomena should correlate better with space-time geometry than with wave mechanics. Number systems in closed form, are only defined in space of 1, 2, 4 or 8 dimensions, known as linear, complex, quaternion and octonion space respectively [22]. All physical evidence suggests that the universe exists in four-dimensional curved space, locally perceived as three-dimensional tangent space, in line with uncritical casual observation. This is akin to the apparent flat-earth geometry on the curved surface of the planet. Any physical model that fails to take all dimensions into account must produce a distorted picture of reality. Molecular matter exists in four-dimensional space and therefore cannot be described correctly in terms of a three-dimensional theory. Self-similar symmetry is a feature of four-dimensional space and connects all structures in the cosmos, without describing any of their intrinsic details. We conjecture that only by solving equation (2) without separation of the variables can a quantum-mechanical model of molecular shape be formulated. However, only a three-dimensional projection is observable in tangent space. This awareness stems from the observation that atoms, molecules, covalence, botanical phyllotaxis, zoological skeletons, solar systems, globular clusters and spiral galaxies are related in self-similar fashion by the symmetry of space time as perceived in tangent space.

#### Space-time Geometry

The geometry of space-time is often simplified and routinely presented in two-dimensional Minkowski space, ${\text{M}}^{2}$. Compared to Euclidean geometry that only has one type of non-zero vector, in ${\text{M}}^{2}$ there are three, depending on the value of the dot product: *A*_1_ · *A*_2_ = −*c*^2^*t*_1_*t*_2_ + *x*_1_*x*_2_, called spacelike if *A*_1_ · *A*_2_ > 0, lightlike if *A*_1_ · *A*_2_ = 0 and time-like if *A*_1_ · *A*_2_ < 0. It is of special interest to note that the distance between any pair of lightlike points, $|A|=\sqrt{A_{1}\cdot A_{2}}=0$, is zero.

In quantum systems defined by (2), although more complicated, a similar counterintuitive situation prevails and intramolecular distances can therefore not be specified in Euclidean measure. This means that the very concept of bond length is quantum-mechanically undefined, which confirms the earlier conclusion that molecular structure is a strictly classical concept.

The behaviour of quantum systems that emerges from this analysis is much more non-classical than traditionally assumed. Direct interaction is not limited to occur between first-neighbour pairs, but operates among all atoms, as could be inferred from non-local interaction mediated by the quantum potential of Bohmian mechanics.

#### 4D Crystallography

The familiar three-dimensional structure is a projection of the four-dimensional shape which is generated as a function of the molecular environment. Structures obtained by crystallographic analysis are the most familiar. As pointed out before [23] crystallographic analysis is also conditioned by the assumption of three-dimensional translational symmetry. To quote:

“. . . the electron-density transform, formulated in terms of space coordinates only, would always project a static ensemble average, even from a situation that fluctuates periodically at the unit cell level. Such fluctuation would add a fourth element of translational symmetry in the time coordinate, but which remains undetected unless a coherent radiation source is used to record a diffraction pattern as a function of time”.

This statement largely anticipates the conclusions reached here on more fundamental grounds.

The molecular structure that emerges is radically different from crystallographic structures. The idea that atoms in a crystal vibrate around fixed positions falls away, to be replaced by an arrangement that fluctuates periodically with time. This non-classical arrangement is conveniently pictured in ${\text{M}}^{2}$.

**Figure:**
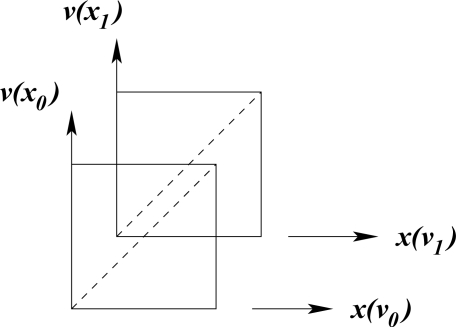


Label the complex coordinate axis *ict* as *v*. The effect of time is modelled by a shift of the unit cell origin. An atom at position *a*(*x*, *v*) appears to wander through the unit cell and returns to the same position in the cell after *h* cycles of *x* and *k* cycles (in another context this is known as a Poincaré recurrence: Every closed dynamic system reverts in time towards its previous state) of *v*. As the isotropic Minkowski diagonal of the cell moves with respect to atomic positions in the cell, the latter appear to fluctuate from time-like to space-like with respect to the origin. At the same time the separation between any two atoms fluctuates continuously from being space-like, through light-like to time-like and this fluctuation is never in phase for any two pairs. This is typical quantum behaviour.

The situation is the same in four-dimensional hypercomplex space ℍ^4^, which we recognize as the essential quantum system, with four, instead of two indices of periodicity. In projection along *v* the geometry changes to the classical ℝ^3^ and all quantum effects disappear.

#### The Quantum Potential

It is instructive to note that by substituting a wave function in complex polar form

$$\Psi =R\;e^{iS/\hbar }$$

into Schrödinger’s equation

$$\frac{\partial \Psi }{\partial t}=\left(-\frac{i\hbar }{2m}\nabla^{2}+V\right)\Psi$$

the real and imaginary parts separate into a quantum Hamilton–Jacobi equation

(3) $$\frac{\partial S}{\partial t}+\frac{{\left(\nabla S\right)}^{2}}{2m}-\frac{\hbar^{2}\nabla^{2}R}{2mR}+V=0$$

and an equation of continuity

$$\frac{\partial R^{2}}{\partial t}+\nabla \left(\frac{R^{2}\nabla S}{m}\right)=0$$

Equation (3) differs from the classical H–J equation only in the term

$$V_{q}=-\frac{\hbar^{2}\nabla^{2}R}{2mR},$$

which Bohm [24] identified as a quantum potential energy to distinguish between classical and non-classical systems. It has the unusual property of showing the amplitude *R* in both numerator and denominator, which is interpreted to mean that quantum interactions operate non-locally, *i.e*., instantaneously over large distances. Exactly this type of interaction is inferred to occur within a four-dimensional quantum molecule between sites in space-like relationship.

The unit charge associated with an electron is defined as

$$e\int_{-\infty }^{\infty }R^{2}(x)dx$$

where the charge density at a point, ρ(*x*) = |*R*^2^(*x*)|. The quantum potential depends on the wave function over all space,

$$V_{q}=-\frac{\hbar^{2}}{2m}\int_{-\infty }^{\infty }\frac{\nabla^{2}R(x)}{R(x)}dx$$

which is a continuous function. The quantum potential energy associated with a pair of hypothetical sub-electronic charge elements *V_q_*(*x*_1_, *x*_2_) is independent of the coordinates of these elements and depends holistically on the quantum state of the whole system. There is no three-dimensional distance-related interaction between charge elements and hence nothing that corresponds to the self-energy of quantum electrodynamics.

A quantum molecule shares many attributes with an electron, with the difference that the charge distribution is discreet, rather than continuous, and of two types—negative and positive. The total molecular wave function is a product function $\psi =\Pi_{i}^{n}\phi_{i}(x_{i},t)$ and the quantum potential is the sum over *n* terms:

$$V_{q}=-\frac{\hbar^{2}}{2}\sum_{i=1}^{n}\frac{\nabla_{i}^{2}R_{i}(x_{i},t)}{m_{i}R_{i}(x_{i},t)}$$

In contrast to the holistic electron, a molecule is said to constitute a partially holistic system.

#### The Quantum Molecule

A quantum molecule has four-dimensional structure which disappears, together with the phase factor, on projection into three-dimensional classical space. There are no chemical bonds in a quantum molecule. It responds holistically to any local distortion and has the ability to rearrange spontaneously *via* intermediates that may appear to be sterically impossible in three dimensions. The internal wave structure of a molecule is periodic and the time-average projection is observed as connectivity in Euclidean space. It is particularly sensitive to photochemical distortion that affects the angular momentum of electron waves. A final complication which has been ignored is the effect of space-time curvature on molecular structure.

#### Space-Time Topology

An additional factor that affects the growth of self-similar structures in Nature is the topology of space-time. Although not known in detail there is sufficient evidence from general relativity to confirm that space-time is topologically closed and most probably of projective geometry [25]. An immediate implication is that the structure of molecules in free space is defined in four-dimensional non-Euclidean space-time. Any classical structural parameter that varies with molecular flexibility should stabilize in free space at a value commensurate with space-time topology. We propose that this equilibrium argument is responsible for cosmic self-similarity, which appears to be related to the golden logarithmic spiral, *r* = *a*e(θτ/2), with homothetic points separated by θ = 2τ, such that Δ*r* = *a*e(*n*πτ). This provides powerful evidence that the curvature of hypercomplex space-time is a function of the three fundamental irrational numbers *e*, π and τ, and $\sqrt{-1}$: *e* for growth, π for rotation and τ for dilatation.

## 3. Conclusions

The difference between quantum and classical structure is now becoming obvious. The quantum world is the four-dimensional aspect of reality which is only partially sensed in the three-dimensional projection perceived in tangent Euclidean space. Quantum objects which appear as wave structures in four-dimensional curved space are observed as three-dimensional particles in tangent space.

The same distinction occurs in optics. There is no duality between the waves of physical optics and the rays of geometrical optics. Likewise, is there no duality between quantum waves and classical particles. It is a question of close and superficial scrutiny. It makes no sense to analyze bulk materials in quantum detail. In the same way that geometrical optics gives a satisfactory account of reflection, focus, refraction and dispersion of a light beam, so does classical mechanics give an adequate account of three-dimensional molecular structure, without going into four-dimensional quantum detail. Confusion sets in when classical methods are used to simulate quantum events, or *vice versa*. This is the reason why *Quantum Chemistry* fails.

The same effect is responsible for creating the delusion of an expanding universe. As explained by Segal [26] the frequency of galactic light in, what he calls, unitime of curved space is represented by the energy operator −*i*(∂/∂τ), but is observed in local flat space, represented by a different operator, −*i*(∂/∂*t*), after decomposition into tangent space and time variables. The implied redshift has nothing to do with expansion.

We can now return to cosmic self-similarity. The mystery disappears on realizing that there is no classical–quantum limit and that quantum theory is not confined to sub-atomic events, but remains valid at all levels. All objects that grow in four-dimensional curved space-time share the same quantum imprint, the only evidence of which, that remains in three-dimensional projection is the self-similar symmetry. It becomes less surprising to find that satellite orbits in the solar system obey quantum rules [14] or to learn about quantized redshifts [27].

### 3.1. Kolbe and van’t Hoff

When Jacobus van’t Hoff first proposed that molecules have rigid three-dimensional structures he was ridiculed by the great chemists of the world, especially Hermann Kolbe, who, as editor of Journal für praktische Chemie, was known to be severely, and sometimes tastelessly, critical of the work of others. Comparing their points of view it could now be argued that Kolbe appreciated the four-dimensional nature of molecules and saw the work of van’t Hoff as a shallowing of this insight.

It was the “supernatural explanation” of optical activity that Kolbe rejected [28]. About thirty years later van’t Hoff was awarded the first Nobel prize in chemistry for this “fanciful nonsense”, which is still repeated in modern textbooks, although dignified by more scholarly-sounding jargon. Now Kolbe is the one who is ridiculed as the old man who refused to acknowledge the theory of two “unknown” scientists.

Instead of looking for evidence that polarized light interacts with a molecular magnetic moment, theoreticians are still agonizing over a quantum-mechanical simulation of molecular chirality and trying to explain how chirality rotates the plane of polarization. It cannot be done. Fact is that molecular chirality is a three-dimensional manifestation of the residual orbital angular momentum, which is described by a complex eigenfunction, and carries the magnetic moment that interacts with polarized light [12]. However, orbital angular momentum in four dimensions, as described by a hypercomplex wave function, is not oriented in the same sense as a three-dimensional vector, and therefore not commensurate with the chiral structure of molecules. It is well established that absolute structure and the direction of optical rotation are not reciprocally related. In the process, known as Walden inversion, it is even possible to change the absolute configuration at an asymmetric carbon, without affecting the direction of rotation. It is remarkable how little comment is excited by the observation that half of the L-amino acids in Nature are *dextro* rotatory.

The van’t Hoff model therefore does not *explain* optical activity, it only serves as a diagnostic. Kolbe’s gut feeling was right.
